# Behavior of self-inflicted violence in patients with bipolar disorder: A systematic review protocol

**DOI:** 10.1097/MD.0000000000016851

**Published:** 2019-08-16

**Authors:** Rita de Cássia Hoffmann Leão, Frederik L. Filho, Carolina M. Fiamoncini, Renata Alves de Sousa, Selene Cordeiro Vasconcelos, Everton Botelho Sougey, Tatiana de Paula Santana da Silva

**Affiliations:** Neuropsychiatry and Behavioral Sciences, Federal University of Pernambuco, UFPE, Recife, Pernambuco, Brazil.

**Keywords:** bipolar disorder, self-injurious behavior, self-mutilation, suicide, suicide attempt

## Abstract

**Background:**

Studies aimed at understanding the higher risk profiles for self-inflicted violence in individuals with BD become essential as a possible predictive risk measure for the presence of suicidal behavior, corroborating the expressive reduction of suicide deaths in young people who are in psychic suffering.

**Methods:**

The protocol was constructed in accordance with Preferred Reporting Items for Systematic Reviews and Meta-Analyzes (PRISMA-P) and the research question was constructed using guidelines from the Population Intervention Comparator Outcome Setting (PICOS) strategy. A third reviewer will be contacted, and two studies will be included in the selection, analysis and inclusion phases of the articles, in case of divergence, a third reviewer will be contacted. (1) methodological design studies of cohorts, case-control and cross-sectional; (2) Diagnosis of Bipolar disorder according to *Diagnostic and statistical Manual of mental disorders V*; (3) Studies with adult population and (4) Studies that consider at least one type of self-inflicted violence as a variable. The articles considered eligible will be analyzed by New Castle - Ottawa quality assessment scale/cross section studies (NOS) to evaluate the quality of the studies.

**Results:**

The identification of the characteristics of self-harm may subsidize professionals who work in the treatment of bipolar disorder with greater attention to these practices and monitoring of possible suicidal behaviors.

**Conclusion:**

This study may represent one of the initial measures of evaluation on these correlations, which will allow to protocol the guidelines in the field of practice and contribute to improvements in public health indexes.

## 1 Introduction

Major mood disorders, specifically bipolar disorder (BD), and major depressive disorder are some of the most prevalent but undiagnosed health problems in children and adolescents.^[1,2]^

The disorder, according to *Diagnostic and Statistical Manual of Mental Disorders, Fifth Edition* (*DSM-V*)^[3]^ differs in 2 main types: type I, in which episodes of mania occur, and type II, in which the elevation of mood is milder and brief, characterizing episodes of hypomania.^[4]^

At present, BD has been associated as one of the highest risk factors for suicidal behavior. Estimates indicate that 25% to 50% of individuals will make one or more attempts at suicide and about 15% will intentionally end their lives.^[5]^

Despite significant research efforts to understand the presence of suicidal behavior in individuals with mood disorders, the diagnosis and evaluation of other risk behaviors such as self-mutilation and other self-inflicted violence practices are still not well understood and addressed during BD treatment.^[6,7]^

In this sense, studies aimed at understanding the higher risk profiles for self-inflicted violence in individuals with BD become essential as a possible predictive risk measure for the presence of suicidal behavior, corroborating the expressive reduction of suicide deaths in young people who are in psychic suffering.

Therefore, there is a need to establish what types of self-injurious behaviors are most evidenced by patients with BD. This will make it possible to protocol the guidelines in the field of practice and contribute to improvements in public health indexes.

There is still no consensus on the correlations under study. This review proposes to create this panorama through the following research question: What are the main behaviors of self-inflicted violence are present in patients with BD?

## 2 Methods

### 2.1 Design and registration of the study, literature search strategy, and study selection

**Figure 1:**
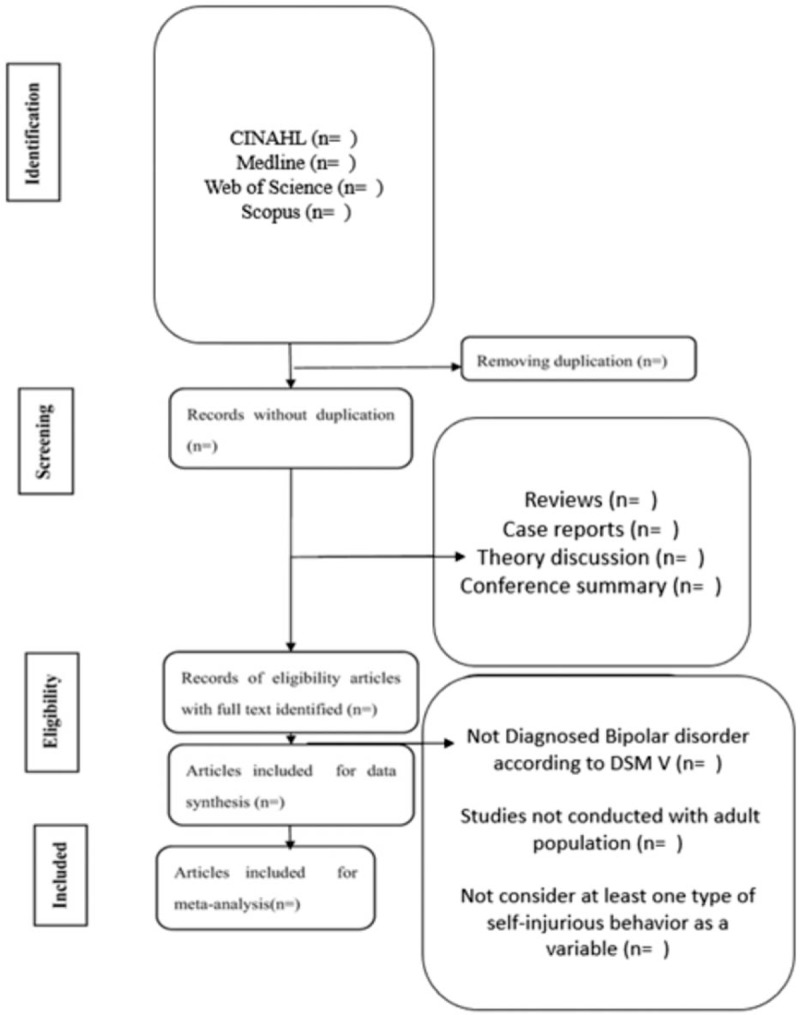
Flow diagram for the protocol.

The proposed revision was duly recorded in the International Prospective Register of Systematic Reviews under protocol: CRD42018086837 (electronic address of record example: https://www.crd.york.ac.uk/prospero/). The methods of the review protocol were written according to Preferred Reporting Items for Systematic Reviews and Meta-Analysis Protocols (PRISMA-P).^[8]^ For the systematic review article, the guidelines of the PRISMA^[9]^ will be followed. The PRISMA-P checklist is available online as supplemental material (additional file 1). The systematic review will be reported according to PRISMA guidelines. Figure 1 reports the flow diagram for the protocol. The guiding question: “What are the main behaviors of self-inflicted violence present in patients with BD?” was created based on the Population Intervention Comparator Outcome Setting^[10]^ guidelines.

The research will be conducted in the electronic databases: CINAHL, Medline via PubMed, Web of Science, and Scopus. After the search, the snowball strategy for data recovery^[11]^ will be adopted. Gray literature will not be considered.

The descriptors registered in Mesh will be considered: “Bipolar disorder”; “Self-mutilation”; “Self-injurious behavior”; “Suicide”; “Suicide attempt,” combined with the Boolean operators “AND” and “OR.” Editorials, news, and letters will be excluded. No limits in the year or language will be applied.

### 2.2 Outcome measures and data extraction

The following studies that will be included will be the ones that: present methodological design studies of cohorts, case-control, and cross-sectional; diagnosed BD according to DSM-V; studies conducted with adult population; consider at least 1 type of self-injurious behavior as a variable. There will be no restrictions on the sex, ethnicity of the participants, or the publication date and language of the article. Review studies and editorial notes will be excluded from the analysis.

The main exposures of interest are the types of self-inflicted violence practiced by individuals with BD. Thus, studies reporting the practice of self-mutilation, suicidal behavior, self-harm, and other self-defeating behaviors will be included in the review. We intend to evaluate the following primary and secondary outcomes of interest:

1. **Primary outcomes:** Studies will be included in the review if the self-inflicted violence variable is cited as a behavior related to BD. The self-inflicted violence can be determined by conducting an interview (self-report), a validated measurement instrument, or a clinical analysis of the health professional.
2. **Secondary outcomes:** The data obtained will be condensed and organized into tables created specifically for the review that will bring the following information:
  a. Characteristics of the study: authors, year of publication, study design, study period.
  b. General characteristics of the sample: characteristics of the population studied (diagnosed with BD), methods of recruitment and sampling, inclusion/exclusion criteria.
  c. Self-harming behavior violence: description of the type of behavior of self-inflicted violence carried out by the individual.

We will consider contacting the corresponding authors for any missing information using a standardized email template.

In the initial phase of screening, 2 authors will independently select the articles through the title analysis and the summary according to the eligibility criteria. These same researchers will do the critical evaluation of the full text of the articles.

To reduce the risk of bias and lack of potentially relevant studies, researchers will take a more forgiving approach at the first screening level. Both researchers will obtain full-text articles for studies that meet the inclusion criteria for the review.

The level of agreement between the 2 reviewers will be calculated, the reasons for the rejection of articles during the initial screening and in the full text evaluation process will be noted and stored in the database. Any discrepancies will be discussed and resolved by a third-party appraiser.

### 2.3 Risk quality assessment of bias quality assessment (in individual studies)

In the evaluation of risk of bias all the articles included will be submitted to analysis of their methodological quality, following New Castle protocol-Ottawa quality assessment scale (NOS)/ cross-section studies.^[12]^ This tool analyzes articles according to selection (sample representativeness, sample size, subjects who did not respond, exposure/risk factors) and comparability and outcomes (evaluation of outcomes, statistical analysis). The qualitative analysis of the NOS is performed using the total classification of 10 stars, distributed in 3 domains: selection (maximum 5 stars), comparability (maximum 2 stars), and outcomes (maximum 3 stars).The strength of evidence using the Grading of Recommendations Assessment, Development and Evaluation approach,^[13]^ data extraction and quality assessment will be performed independently by 2 authors. The information will be examined and judged independently by a third author when necessary.

### 2.4 Data synthesis

The data obtained will be exported to Endnote V.7.1 and duplicate records will be removed electronically. Tracing and extraction will occur in a database created specifically for review in Microsoft Excel to ensure that all retrieved references are fully tracked.

### 2.5 Ethics and dissemination

This systematic review does not require ethical approval and informed consent, and the outcome will be released as a literature review and conference for the clinician.

## 3 Discussion

Despite the current pressure on health, legal, and social services systems, research and assistance in the field of self-inflicted violence throughout the world is still incipient, especially in the psychiatric comorbidities involved in this dynamic.

Particularly DB has been associated as one of the major risk factors for self-inflicted violence behaviors, especially suicidal behavior. Thus, it is necessary to establish which types of self-injurious behavior are most evidenced by patients with Bipolar disorder. Thus, this study may represent one of the initial measures of evaluation on these correlations, which will allow to protocol the guidelines in the field of practice and contribute to improvements in public health indexes.

## 4 Limitations

The main limitations of the study include

- Noninclusion of gray literature
- The investigation will provide the first evidence on the profile of self-inflicted violence practiced by bipolar patients
- By publication it will be possible to clarify which are the main behaviors of self-inflicted violence practiced by patients with BD
- The publication will use quantitative methods to evaluate the strength of the evidence found
- Study developed in a single research center

## Author contributions

**Conceptualization:** Rita Cássia Hoffmann Leão, Renata Alves de Sousa, Selene Cordeiro Vasconcelos.

**Formal analysis:** Tatiana de Paula Santana da Silva.

**Investigation:** Rita Cássia Hoffmann Leão, Frederik Lapa Filho, Selene Cordeiro Vasconcelos, Tatiana de Paula Santana da Silva.

**Methodology:** Rita Cássia Hoffmann Leão, Frederik Lapa Filho, Carolina Maciel Fiamoncini, Renata Alves de Sousa, Selene Cordeiro Vasconcelos, Everton Botelho Sougey, Tatiana de Paula Santana da Silva.

**Project administration:** Frederik Lapa Filho, Tatiana de Paula Santana da Silva.

**Supervision:** Rita Cássia Hoffmann Leão, Selene Cordeiro Vasconcelos, Tatiana de Paula Santana da Silva.

**Visualization:** Carolina Maciel Fiamoncini, Renata Alves de Sousa.

**Writing – review and editing:** Rita Cássia Hoffmann Leão, Carolina Maciel Fiamoncini, Renata Alves de Sousa, Selene Cordeiro Vasconcelos, Everton Botelho Sougey, Tatiana de Paula Santana da Silva.
